# Antibiogram and Antibiotic Resistance Patterns in Bacterial Isolates from Hayatabad Medical Complex, Peshawar

**DOI:** 10.7759/cureus.71934

**Published:** 2024-10-20

**Authors:** Mehwash Iftikhar, Imran Khan, Sheraz J Khan, Jehan Z Khan, Saeed Ur Rahman

**Affiliations:** 1 Medicine, Hayatabad Medical Complex Peshawar, Peshawar, PAK; 2 Internal Medicine, Hayatabad Medical Complex Peshawar, Peshawar, PAK; 3 Pharmacy, University of Peshawar, Peshawar, PAK; 4 Pathology, Khyber Girls Medical College (KGMC), Peshawar, PAK; 5 Pathology, Hayatabad Medical Complex Peshawar, Peshawar, PAK

**Keywords:** antibiogram, antimicrobial resistance, empirical antibiotic therapy, gram-negative and gram-positive bacteria, multidrug-resistant organisms (mdr)

## Abstract

Background

Empirical antibiotic therapy is facilitated by antibiograms as they provide local bacterial resistance data and patterns. Antibiograms are critical tools that offer comprehensive, institution-specific information on antimicrobial susceptibilities, enabling clinicians to make informed decisions about empirical treatment and guiding antimicrobial stewardship efforts. The rising incidence of multidrug-resistant (MDR) organisms is a significant challenge in countries like Pakistan.

Objective

The objective of this study was to evaluate the resistance patterns of Gram-negative and Gram-positive bacteria at Hayatabad Medical Complex (HMC) to commonly used antibiotics, with a specific focus on identifying MDR pathogens.

Methods

A retrospective study was conducted using antibiogram data from January to September 2022, focusing on both Gram-negative and Gram-positive organisms. Sensitivity patterns for antibiotics such as Ceftazidime, Imipenem, Meropenem, Amikacin, Oxacillin, and Teicoplanin were checked.

Results

*Acinetobacter* posed a significant challenge to treatment, displaying only 6% sensitivity to Ceftazidime. This extremely low sensitivity indicates an alarmingly high level of resistance, which is of great concern as it severely limits treatment options for *Acinetobacter* infections. Such high resistance to Ceftazidime, a broad-spectrum cephalosporin, suggests that infections caused by this pathogen may require the use of last-resort antibiotics or combination therapies, potentially leading to increased healthcare costs and poorer patient outcomes. Additionally, *Acinetobacter* showed only 37% sensitivity to Ciprofloxacin, further confirming its status as an MDR pathogen in hospital settings.

Conclusion

The results show the growing threat of MDR organisms at HMC, Peshawar. This underscores the urgent need for robust antimicrobial stewardship programs, enhanced infection control practices, ongoing surveillance of resistance patterns, healthcare provider education, and regional collaboration to address the challenge of antimicrobial resistance.

## Introduction

Antimicrobial resistance (AMR) is a global health crisis with particularly severe implications for developing countries like Pakistan. The misuse and overuse of antibiotics, coupled with inadequate infection control practices, particularly in overcrowded hospitals with limited resources, also contribute to the spread of resistant pathogens [1]. In Pakistan, this issue is exacerbated by limited resources for comprehensive antimicrobial stewardship programs and the high prevalence of healthcare-associated infections (HAIs).

The increasing resistance among Gram-negative organisms, such as *Escherichia coli*, *Pseudomonas aeruginosa*, and *Acinetobacter baumannii*, presents a significant public health threat. These pathogens are responsible for a range of serious infections, including bloodstream infections, pneumonia, and urinary tract infections, all of which are more difficult to treat due to rising antibiotic resistance [2-4]. This problem is compounded by the fact that many hospitals in Pakistan are overcrowded, lack adequate infection control measures, and struggle to maintain proper hygiene standards, which allows the spread of resistant pathogens.

Antibiograms are a critical tool in addressing AMR, offering essential, local data to guide empirical therapy. By providing a snapshot of current resistance patterns, antibiograms enable clinicians to make evidence-based decisions about antibiotic use, ensuring timely and targeted treatment. In resource-limited settings like Pakistan, where access to more effective, costly antibiotics is restricted, the reliance on antibiograms is crucial for reducing inappropriate therapy, which can exacerbate resistance development [5].

Numerous studies from Pakistan have reported alarmingly high resistance rates to critical antibiotics. For example, research from Karachi and Lahore has shown over 70% resistance in *Escherichia coli *to fluoroquinolones and up to 55% carbapenem resistance in *Acinetobacter baumannii*, respectively [6-8]. These resistance trends reflect global patterns yet are often more severe in developing countries due to unregulated antibiotic use and insufficient healthcare infrastructure.

Despite the global and regional recognition of AMR, there is limited data on resistance patterns from tertiary care hospitals in Pakistan, particularly in Khyber Pakhtunkhwa. This study aims to address this gap by examining the antibiotic resistance profiles at Hayatabad Medical Complex (HMC), one of Peshawar's largest tertiary care hospitals. By comparing local data with regional and national trends, this research seeks to identify whether the resistance patterns at HMC align with broader trends or whether specific local interventions are necessary.

The findings from this study will not only contribute to improved clinical decision-making but will also support the development of targeted antimicrobial stewardship programs. Such efforts are essential for preserving the efficacy of the limited antibiotics that are still effective against MDR organisms in Pakistan, ultimately improving patient outcomes and mitigating the further spread of resistance.

## Materials and methods

Study design and setting

Our retrospective study was conducted at HMC in Peshawar from January to September 2022. HMC was chosen for this study as it is a major tertiary care hospital serving a large population in Peshawar and the surrounding regions. Studying resistance patterns here provides valuable insights into regional trends and can inform antibiotic policies for similar facilities in the area.

Sample collection and processing

We included a total of 2,500 bacterial isolates from clinical specimens (blood, urine, wounds, respiratory samples) that yielded significant growth during the study period. Both outpatient and inpatient samples were analyzed.

Inclusion criteria

- Bacterial isolates from clinical specimens yielding significant growth

- Non-duplicate isolates (first isolate per patient per species)

Exclusion criteria

- Contaminated specimens

- Isolates identified as likely contaminants based on clinical correlation

- Repeat isolates from the same patient

- Isolates with incomplete susceptibility data

These exclusions were made to maintain data integrity and ensure that the antibiogram accurately reflects clinically relevant infections rather than colonization or contamination.

Bacterial identification and antibiotic susceptibility testing

Our laboratory team identified all organisms using standard microbiological methods. Antibiotic susceptibility testing was performed following current Clinical and Laboratory Standards Institute (CLSI) guidelines [4]. We collected data on organism type, specimen source, and susceptibility patterns to commonly prescribed antibiotics at our institution.

Antibiotics tested

These antibiotics were chosen based on their clinical relevance and frequency of use in our setting: for Gram-negative organisms are Ceftazidime, Imipenem, Meropenem, Amikacin, and Ciprofloxacin; for Gram-positive organisms are Oxacillin, Teicoplanin, Vancomycin, Ciprofloxacin, and Levofloxacin.

Statistical analysis

Data were analyzed using SPSS (IBM SPSS Statistics for Windows, IBM Corp., Version 26, Armonk, NY). Descriptive statistics were used to summarize the findings. We calculated sensitivity percentages with 95% confidence intervals for each organism-antibiotic combination. Results were classified as sensitive, intermediate, or resistant based on CLSI breakpoints. 

Ethical considerations

The study was approved by the Institutional Review Board of HMC (Ref: HMC/EC/2022-156). As this was a retrospective study using anonymized laboratory data, the need for individual patient consent was waived. The authors declare no conflicts of interest in relation to this study. This research did not receive any specific grant from funding agencies in the public, commercial, or not-for-profit sectors.

## Results

The results revealed significant resistance trends among both Gram-negative and Gram-positive organisms isolated at HMC.

Gram-negative organisms

Among the Gram-negative isolates, *Escherichia coli* and *Klebsiella* spp. showed moderate resistance to Ceftazidime, with sensitivity rates of 43% and 32%, respectively (Table 1). These organisms, however, demonstrated high sensitivity to the carbapenems Imipenem and Meropenem, both achieving sensitivity rates above 80%. Amikacin was also notably effective, with *Escherichia coli* exhibiting a 93% sensitivity rate, which is encouraging given the organism's propensity for causing urinary tract infections, which is one of the most common infections in the community. *Pseudomonas* and *Acinetobacter* posed a significant challenge to treatment. *Pseudomonas* displayed high resistance to both Ceftazidime (75% resistance) and Ciprofloxacin (47% resistance), and although Imipenem (56% sensitivity) and Amikacin (63% sensitivity) fared better, these figures indicate a declining efficacy of these antibiotics. *Acinetobacter* was the most resistant, with only 6% sensitivity to Ceftazidime and 37% sensitivity to Ciprofloxacin, labeling itself as an MDR pathogen in hospital settings. Carbapenems like Meropenem and Imipenem showed higher efficacy (77% and 65% sensitivity, respectively) against MDR *Acinetobacter*; their continued misuse may exacerbate resistance development in the near future. 

**Table 1:** Sensitivity Patterns for Gram-Negative Organisms (% Sensitive, 95% CI)

Organism	Ceftazidime	Imipenem	Meropenem	Amikacin	Ciprofloxacin
Escherichia coli	43% (39.8-46.2)	82% (79.3-84.7)	82% (79.3-84.7)	93% (91.2-94.8)	65% (61.8-68.2)
Klebsiella	32% (28.4-35.6)	87% (84.4-89.6)	88% (85.5-90.5)	90% (87.7-92.3)	63% (59.3-66.7)
Pseudomonas	25% (21.3-28.7)	56% (51.8-60.2)	51% (46.8-55.2)	63% (58.8-67.2)	53% (48.8-57.2)
Acinetobacter	6% (3.8-8.2)	65% (60.1-69.9)	77% (72.6-81.4)	71% (66.3-75.7)	37% (32.2-41.8)

Gram-positive organisms

The results were comparatively favorable for Gram-positive organisms (Table 2).* Staphylococcus aureus *exhibited high sensitivity to Teicoplanin (85%) and Vancomycin (92%), which are critical agents in the treatment of methicillin-resistant *Staphylococcus aureus* (MRSA) infections. This suggests that, despite rising resistance concerns, these antibiotics remain effective options. *Staphylococcus epidermidis*, another common Gram-positive organism, demonstrated a similar pattern, with high sensitivity to Vancomycin (90%) and Teicoplanin (87%). There was a notable resistance to Oxacillin in both *Staphylococcus aureus* and *Staphylococcus epidermidis* (68% and 60% sensitivity, respectively). Ciprofloxacin and Levofloxacin were less effective against these Gram-positive organisms, with sensitivity rates hovering around 50-60%, suggesting that these antibiotics must not be used as first-liners for Gram-positive infections.

**Table 2:** Sensitivity Patterns for Gram-Positive Organisms (% Sensitive, 95% CI)

Organism	Oxacillin	Teicoplanin	Vancomycin	Ciprofloxacin	Levofloxacin
Staphylococcus aureus	68% (62.3-73.7)	85% (80.5-89.5)	92% (88.5-95.5)	59% (53.1-64.9)	63% (57.2-68.8)
Staphylococcus epidermidis	60% (49.6-70.4)	87% (79.2-94.8)	90% (82.8-97.2)	51% (40.5-61.5)	58% (47.5-68.5)

Overall, the findings highlight an alarming trend of high resistance rates to commonly used antibiotics like Ceftazidime and Ciprofloxacin, particularly in Gram-negative organisms. Carbapenems and aminoglycosides, such as Imipenem and Amikacin, continue to demonstrate efficacy, but the ongoing threat of resistance necessitates their judicious use. In Gram-positive organisms, while Vancomycin and Teicoplanin remain highly effective, the increasing resistance to Oxacillin underscores the persistent risk of MRSA in hospital environments.

These results underscore the urgent need to develop robust antimicrobial stewardship programs. Continuous monitoring of resistance patterns is crucial to guide empirical therapy and prevent the spread of MDR organisms.

## Discussion

This study provides critical insights into the AMR patterns at HMC, particularly focusing on multidrug-resistant (MDR) organisms. The findings highlight the growing resistance to commonly used antibiotics, particularly among Gram-negative bacteria, such as *Acinetobacter* and *Pseudomonas*, which demonstrated high resistance to Ceftazidime and Ciprofloxacin. However, the study’s clinical implications extend beyond these observations and suggest specific changes to empirical treatment guidelines.

First, the high resistance rates to third-generation cephalosporins, such as Ceftazidime, suggest that these antibiotics should no longer be recommended as first-line empiric therapy for Gram-negative infections at HMC. Instead, carbapenems like Imipenem and Meropenem should be prioritized, as they exhibited relatively higher sensitivity rates. However, given the increasing carbapenem resistance observed in both regional and global studies [5-8], it is crucial to implement strict antimicrobial stewardship programs [2-4,6]. These programs should focus on limiting the overuse of carbapenems to preserve their efficacy.

For *Acinetobacter* infections, which showed alarmingly low sensitivity to Ceftazidime (6%) and Ciprofloxacin (37%), more aggressive treatment strategies may be required. Combination therapies or the use of last-resort antibiotics like Colistin should be considered in such cases. In addition, empirical coverage for *Acinetobacter* should prioritize carbapenems, especially Meropenem, given its superior sensitivity (77%). The rising resistance of Gram-negative bacteria to available antibiotics highlights the urgent need for developing new treatment protocols tailored to local resistance patterns.

In the case of Gram-positive infections, particularly those caused by *Staphylococcus aureus*, the high rates of methicillin resistance (MRSA) call for the use of Vancomycin or Teicoplanin as empiric therapies. These antibiotics demonstrated high sensitivity, making them crucial for the management of MRSA infections. However, the growing resistance to Oxacillin (68% sensitivity in *Staphylococcus aureus* and 60% in *Staphylococcus epidermidis*) points to an increasing prevalence of methicillin-resistant strains [9-12]. The persistence of these resistant strains in hospitals is likely due to a combination of factors, including inadequate infection control practices, overuse of antibiotics, and the high transmissibility of MRSA in overcrowded hospital environments [13,14].

To address the increasing resistance patterns observed in this study, antimicrobial stewardship programs at HMC should include several key components. Firstly, a hospital-wide antibiotic restriction policy should be implemented, particularly restricting the use of broad-spectrum antibiotics like carbapenems. A mandatory infectious disease consultation should be required for carbapenem prescriptions exceeding 72 hours to ensure judicious use. Additionally, rapid diagnostic technologies should be introduced to identify pathogens and their resistance profiles earlier, allowing clinicians to initiate targeted therapies more promptly.

Educational efforts are also essential to support the appropriate prescribing of antibiotics. Regular workshops should be conducted to train healthcare providers on interpreting antibiogram data and making informed decisions about empirical therapies. Moreover, continuous surveillance of antibiotic use and resistance patterns should be established, with feedback provided to prescribers on their antibiotic prescribing habits. This feedback loop will be crucial in curbing the overprescription of antibiotics and promoting more responsible use of available drugs.

Finally, improving infection control measures at HMC is paramount to preventing the spread of MDR organisms. Hand hygiene compliance must be strengthened through regular audits and feedback to healthcare workers. Additionally, contact precautions should be implemented for patients infected with MDR organisms to contain their spread. These measures, combined with robust antimicrobial stewardship programs, are critical steps in addressing the growing threat of AMR at HMC.

The limitations of our study include a relatively small sample size, which may restrict the generalizability of the findings. Additionally, the retrospective design posed inherent risks of selection bias and incomplete data, which could influence the validity of the results. The study was also conducted in a single institution, limiting its broader applicability to other settings. Furthermore, we did not collect certain clinical data that could provide a more comprehensive analysis of the resistance patterns. A more extensive, prospective study would help overcome these limitations and offer stronger, more reliable conclusions.

## Conclusions

The antibiogram data from HMC in Peshawar, Pakistan, highlighted significant challenges posed by MDR organisms in hospital settings. The high resistance levels among Gram-negative bacteria, particularly *Acinetobacter* and *Pseudomonas*, underscore the urgent need for improved infection control practices and antimicrobial stewardship initiatives. Carbapenems, specifically Imipenem and Meropenem, demonstrated relatively higher efficacy and are critical for treating MDR infections; however, their use must be carefully managed to prevent further resistance. While the current prevalence of methicillin-sensitive strains of *Staphylococcus aureus* and *Staphylococcus epidermidis* is somewhat reassuring, the risk of methicillin-resistant strains (MRSA) remains a concern. The high sensitivity to Teicoplanin and Vancomycin provides valuable treatment options for resistant Gram-positive infections. Nonetheless, the sustainability of this sensitivity pattern is uncertain, and future antibiograms will be essential in tracking trends and informing treatment guidelines. To address these challenges, we strongly recommend the implementation of a comprehensive antimicrobial stewardship program at HMC, enhancing infection control measures, conducting ongoing surveillance, educating healthcare providers, and engaging in regional collaboration. These actions are crucial to preserving the efficacy of our current antibiotic arsenal and improving patient outcomes, as the threat of MDR organisms requires immediate and sustained action from all stakeholders in the healthcare system.
